# Aligned and realistic latent diffusion for text-to-motion generation

**DOI:** 10.1186/s42492-026-00224-2

**Published:** 2026-07-20

**Authors:** Zhaowu Li, Rui Liu, Deheng Zhu, Dongsheng Zhou, Xiaopeng Wei

**Affiliations:** 1 National and Local Joint Engineering Laboratory of Computer Aided Design, School of Software Engineering, Dalian University, Dalian, Liaoning 116622 China; 2 School of Computer Science and Technology, Dalian University of Technology, Dalian, Liaoning 116024 China

**Keywords:** Text-to-motion generation, Latent diffusion model, Contrastive learning

## Abstract

Text-to-motion generation aims to synthesize semantically consistent and naturally coherent motion sequences from natural language descriptions. Given the continuous nature of human motion, diffusion models operating in a continuous latent space offer inherent advantages over vector quantization-based methods, particularly in avoiding quantization errors and in modeling quality. However, existing diffusion models primarily rely on mean squared error loss. This stepwise regression paradigm often leads to ‘over-smoothed’ motion sequences and struggles to capture the subtle semantic nuances embedded in textual descriptions. To realize the potential for continuous diffusion generation, an enhanced latent-space diffusion framework designed to elevate generation capabilities across two dimensions, namely, distribution approximation and semantic alignment, is proposed. Specifically, a latent-space adversarial discriminator is incorporated. By applying decoupled adversarial supervision, this component mitigates the detail loss caused by mean regression, significantly enhancing the physical realism and dynamic sharpness. Concurrently, a latent-space contrastive alignment strategy is introduced during the denoising process that reinforces the correspondence of the generated motion sequences with the given textual inputs via explicit cross-modal constraints. Extensive experiments on standard benchmarks demonstrate that the proposed method effectively addresses the limitations of conventional diffusion models, thus validating the potential of continuous diffusion frameworks within the domain of text-driven motion synthesis.

## Introduction

Text-to-motion generation aims to synthesize semantically consistent, structurally plausible, and naturally coherent 3D human motion sequences from natural language descriptions. This technology offers an efficient solution for content creation in applications such as character animation, intelligent interactions, film production, and game development. However, this task presents significant challenges, because it requires the model to accurately comprehend textual semantics and also ensure that the synthesized motions exhibit realistic dynamic structures, spatial consistency, and fine-grained details. This is challenging in both cross-modal alignment and temporal sequence modeling.

The inherent cross-modal discrepancy between text and motion also poses significant challenges in text-to-motion tasks. Although textual descriptions typically convey rich semantic information, motion data exist in high-dimensional spatiotemporal sequences. Thus, the effective bridging of the semantic gap between these two modalities remains a critical issue. A mainstream category of approaches (e.g., T2M [1], TM2T [2], MotionCLIP [3]) addresses this issue by representing continuous motion data as discrete latent vectors and learning the motion distribution within this latent space. Although these methods achieve promising results in text-semantic consistency, the discretization of motion representations inevitably leads to the loss of continuous motion information. Consequently, the generated motions often suffer from insufficient temporal smoothness and a lack of fine-grained details, thus failing to accurately reflect the natural dynamic characteristics of human motion.

In recent years, inspired by the success of diffusion models in image and video generation, researchers have leveraged their powerful stepwise denoising mechanisms for human motion generation. Representative works such as MDM [4], MotionDiffuse [5], and ReMoDiffuse [6] have demonstrated the potential of diffusion models in raw motion space. MLD [7] introduced a latent diffusion paradigm to address the high computational costs, striking an optimal balance between inference efficiency and generative quality. Building on this foundation, recent studies have enhanced the flexibility and controllability of latent diffusion. ReMoDiffuse [6] introduced external knowledge via retrieval augmentation, whereas MotionMamba [8] utilized state space models instead of transformers to achieve linear complexity for long-sequence modeling.

Compared with discrete vector quantization (VQ) approaches, latent-space diffusion methods offer a more streamlined training and sampling pipeline, effectively circumscribing the information loss induced by quantization errors. Nevertheless, diffusion models, whether operating in raw data or latent spaces, consistently exhibit a significant performance gap in terms of generation quality compared to state-of-the-art (SOTA) VQ-based methods. This disparity has motivated a critical reexamination of the fundamental limitations of diffusion models within this domain. Recent studies, such as MARDM [9] and MMDM [10] have highlighted constraints related to model architectures and the challenges of modeling complex motion distributions. Crucially, the authors identify that the current performance bottleneck of latent diffusion models (LDMs) converges on an often-overlooked deficiency: the inherent limitations of the standard mean squared error (MSE) training objective.

The standard MSE objective is effective for stepwise noise prediction, but it is insufficient for text-to-motion diffusion models in two aspects. First, MSE loss mainly optimizes local denoising fidelity and does not explicitly enforce semantic consistency between generated motion latents and textual descriptions. As a result, the generated motions may be physically plausible but semantically weakly aligned with the input text. Second, the regression nature of MSE tends to drive the model toward averaged solutions, which may suppress high-frequency motion details and reduce dynamic sharpness. This over-smoothing effect weakens the realism and diversity of generated human motions, leading to suboptimal performance on perceptual metrics such as Fréchet inception distance (FID).

Adversarial training and cross-modal alignment provide useful inspiration for addressing the above limitations from two complementary perspectives. Adversarial training has established itself as a cornerstone of generative modeling, with wide applications in image, video, and motion synthesis. Classic generative adversarial networks (GANs) employ a minimax game between the generator and discriminator, which enhances realism, detail preservation, and high-frequency structural fidelity. In the image domain, approaches such as diffusion-GAN [11] and E-LatentLPIPS [12] have explored the use of discriminator signals as auxiliary supervision to steer the diffusion process toward authentic data distributions. Similarly, in video and motion-related generation tasks, methods such as MagicAnimate [13] and GENMO [14] leverage temporal or motion-aware discriminators to improve dynamic consistency. For human motion synthesis, GAN-based methods, including Text2Action [15], MoDi [16], and LS-GAN [17], have demonstrated the effectiveness of adversarial supervision in improving spatial naturalness and motion realism. These studies indicate that adversarial feedback can complement reconstruction-based objectives by providing explicit constraints on the generated distribution. However, existing text-to-motion diffusion methods, such as MDM [4], MLD [7], and MARDM [9], still primarily rely on MSE-based denoising supervision and often lack direct feedback from the true motion distribution. This may lead to over-smoothed dynamics, motion drift, unnatural poses, or kinematic inconsistencies.

In parallel, contrastive learning has become an effective strategy for cross-modal representation alignment. By pulling matched pairs closer and pushing unmatched pairs apart, contrastive objectives help models learn structured semantic relationships across modalities. Models such as CLIP have demonstrated the effectiveness of this paradigm in image-text alignment, and information noise-contrastive estimation (InfoNCE)-based objectives have become a common choice for multimodal representation learning. In motion generation, MotionCLIP [3] aligns the motion manifold with the contrastive language-image pre-training (CLIP) space to enable text-guided motion synthesis, whereas TMR [18] improves the earlier TEMOS [19] framework by introducing contrastive supervision for text-motion retrieval. More recent studies, such as MotionBind [20] and KinMo [21], further extend alignment learning to multimodal or hierarchical motion representations. In addition to static representation alignment, several methods introduce alignment constraints into the generation process. For example, ReMoDiffuse [6] uses a retrieval module trained with contrastive learning to provide semantically similar motion references, and HUMAN-TOMATO [22] employs an external alignment network to guide motion synthesis. Although these methods improve semantic consistency, they either focus mainly on static representation spaces or rely on external alignment modules. Therefore, how to directly impose semantic alignment constraints within the latent diffusion denoising process remains insufficiently explored.

To address these challenges, a multilevel diffusion optimization framework is proposed. By incorporating discriminative supervision and cross-modal semantic alignment, the proposed approach systematically mitigates the fundamental limitations of existing diffusion models. Ultimately, it significantly enhances both the generation quality and semantic consistency of text-driven human-motion synthesis. The primary contributions of this study are summarized as follows:

1. Latent adversarial training: Drawing inspiration from the success of GANs in image and video synthesis, the authors integrate explicit adversarial supervision into the latent diffusion training pipeline. This mechanism provides feedback on the authentic motion distribution, compelling the model to go beyond minimizing reconstruction error to actively approximate the underlying data manifold. Consequently, this enhances the naturalness and dynamic fidelity of the generated motions.
2. Temporal semantic contrastive learning (TSCL): Leveraging the principles of contrastive learning widely used in multi-modal pre-training (e.g., CLIP), the authors introduce an InfoNCE-based objective within the latent space to align text and motion representations. This objective function directly encourages alignment between the learned motion representations and their associated textual semantics, thereby reinforcing semantic consistency and cross-modal synergy.
3. Extensive experiments carried out using the HumanML3D and KIT-ML benchmarks with empirical evidence demonstrate that the proposed approach achieves performance comparable to or surpassing SOTA VQ-based methods, validating the substantial potential of diffusion frameworks for text-driven motion generation.

Compared with existing approaches, the proposed method achieves an optimal balance between semantic consistency and motion quality by effectively integrating adversarial optimization with cross-modal alignment. This offers a novel paradigm for high-quality text-driven human-motion generation.

## Methods

### Problem definition

**Fig. 1:**
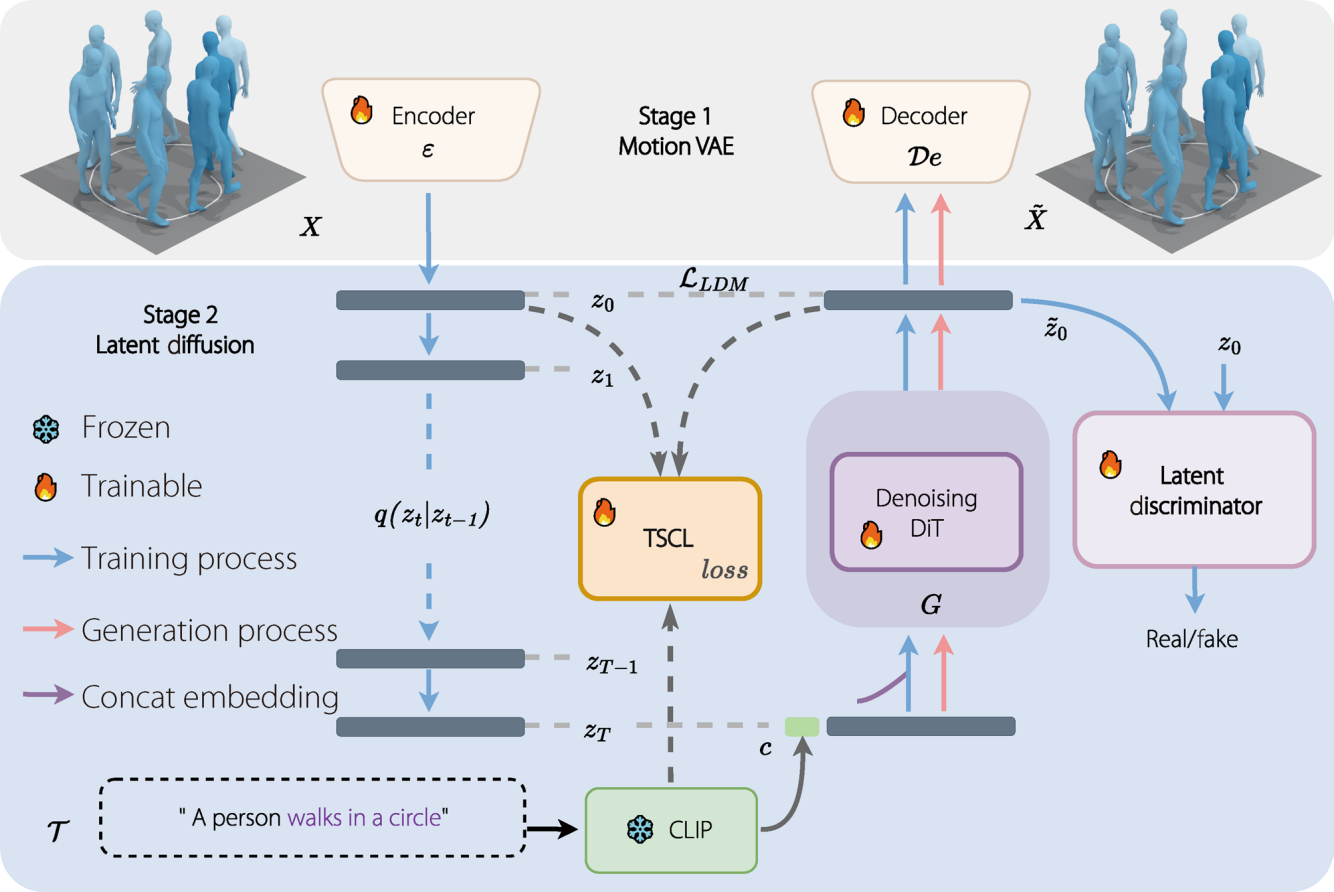
Overview of the proposed framework. A pre-trained variational autoencoder is utilized to establish a continuous latent space $\mathcal{Z}$. The core contribution of this study lies in the diffusion generation phase, where an enhanced diffusion transformer is trained within this latent space. (1) the authors incorporate a latent-discriminator to enable adversarial training for enhanced realism; and (2) enforce a contrastive loss (TSCL) to strengthen the text-motion alignment. VAE: Variational autoencoder; TSCL: Temporal semantic contrastive learning; DiT: Diffusion transformer; CLIP: Contrastive language-image pre-training

A unified framework based on continuous latent-space diffusion is proposed to generate high-fidelity human motions with strong semantic grounding in natural language. Let $\mathcal{T} = \{t_1, t_2, \dots, t_N\}$ represent a natural language description comprising $N$ word tokens. The task is to produce a temporally coherent motion $\boldsymbol{X} = [\boldsymbol{x}_1, \boldsymbol{x}_2, \dots, \boldsymbol{x}_M] \in \mathbb{R}^{M \times D}$, where $M$ corresponds to the number of motion frames and $D$ denotes the dimensionality of the pose representation. The synthesized sequence $\boldsymbol{X}$ must satisfy two key criteria. It should be semantically aligned with the text $\mathcal{T}$ and structurally plausible with temporal coherence. As shown in Fig. 1, the proposed approach follows a two-stage generation paradigm.

#### Motion representation and latent space

The authors construct a variational autoencoder (VAE) using a 1D convolutional architecture [23]. It comprises an encoder $\mathcal{E}$ and decoder $\mathcal{D}e$, both utilizing 1D convolutions with residual connections. This design aims to map high-dimensional complex human motion data into a compact, low-dimensional, and continuous latent representation $\mathcal{Z}$. To ensure high-quality latent representations and reconstruction fidelity, a joint optimization strategy that combines reconstruction loss, Kullback-Leibler divergence, and adversarial loss is employed. In contrast to discrete VQ-based approaches, a continuous latent space offers superior dynamic expressiveness while circumventing quantization errors, striking a favorable balance between the generation quality and inference efficiency. Upon completion of the training stage, the VAE parameters are frozen. The subsequent generation process operates entirely within this compact latent space $\mathcal{Z}$, allowing the diffusion model to concentrate on a high-level semantic alignment, thereby significantly enhancing both generation quality and efficiency.

#### Latent motion diffusion model

In the second stage, the objective is to synthesize the corresponding latent motion representations conditioned on the text representations. A conditional LDM is employed [23]. Diffusion models operate by iteratively corrupting data with noise via a forward process and subsequently training a denoising network to reverse this trajectory, thereby enabling the synthesis of high-quality motion from pure noise. The forward process is formulated as a fixed Markov chain. Gaussian noise is gradually added to the clean motion latents $\boldsymbol{z}_0$ until the signal degrades into an isotropic standard normal distribution $\boldsymbol{z}_T \sim \mathcal{N}(\boldsymbol{0}, \boldsymbol{I})$. The single transition step is defined as follows:

1 $$q(\boldsymbol{z}_t | \boldsymbol{z}_{t-1}) = \mathcal{N}(\boldsymbol{z}_t; \sqrt{1 - \beta_t} \boldsymbol{z}_{t-1}, \beta_t \boldsymbol{I})$$

Here, $\boldsymbol{z}_t$ denotes the noisy latent state at timestep $t$ and $\beta_t$ represents a predefined noise schedule controlling the intensity of the noise injection. A denoising network $\epsilon_\theta(\boldsymbol{z}_t, t, c)$ predicts the noise residuals at each timestep, given noisy latent inputs, diffusion index $t$ and text-derived condition $c$. Here, $c$ denotes text embeddings extracted using a pretrained CLIP encoder. The optimization objective is standard noise prediction loss as follows:

2 $$\mathcal{L}_{LDM} = \mathbb{E}_{\boldsymbol{z}_0, t, c, \boldsymbol{\epsilon}} \left[ \| \boldsymbol{\epsilon} - \boldsymbol{\epsilon}_\theta(\boldsymbol{z}_t, t, c) \|_2^2 \right]$$

While this baseline model generates plausible motions, relying solely on the MSE loss often leads to the ‘over-smoothing’ problem. To address this issue, a unified framework is proposed (Fig. 1) that incorporates two key components as described below. (1) Adversarial motion discriminator: A discriminator to provide explicit supervision on generation quality is introduced. Enforcing the model to approximate the true motion distribution via adversarial learning compels the generator to capture richer details, thereby enhancing global naturalness and dynamic plausibility. (2) TSCL: A contrastive learning strategy is directly integrated into the diffusion training pipeline. By maximizing the semantic correlation between the motion representations (both real and generated) and textual representations, this mechanism continuously reinforces semantic constraints throughout the denoising phase.

### Adversarial motion discriminator

Despite the remarkable capabilities of diffusion models in generating smooth and diverse motions, their training primarily relies on a simple MSE loss. This stepwise reconstruction paradigm tends to produce ‘averaged’ sequences, often characterized by over-smoothing and a loss of fine-grained details, which can reduce both the consistency and physical plausibility. Inspired by recent progress in adversarial training (e.g., MoDi [16], LS-GAN [17], MoLA [23]), the authors introduce an adversarial motion discriminator $D_\phi$ within the latent space. Unlike traditional discriminators operating in a raw pose space, the proposed module works directly on latent representations. This approach avoids the extra cost of decoding while leveraging the compact semantic representations of the latent features, thereby facilitating a more stable training.

**Fig. 2:**
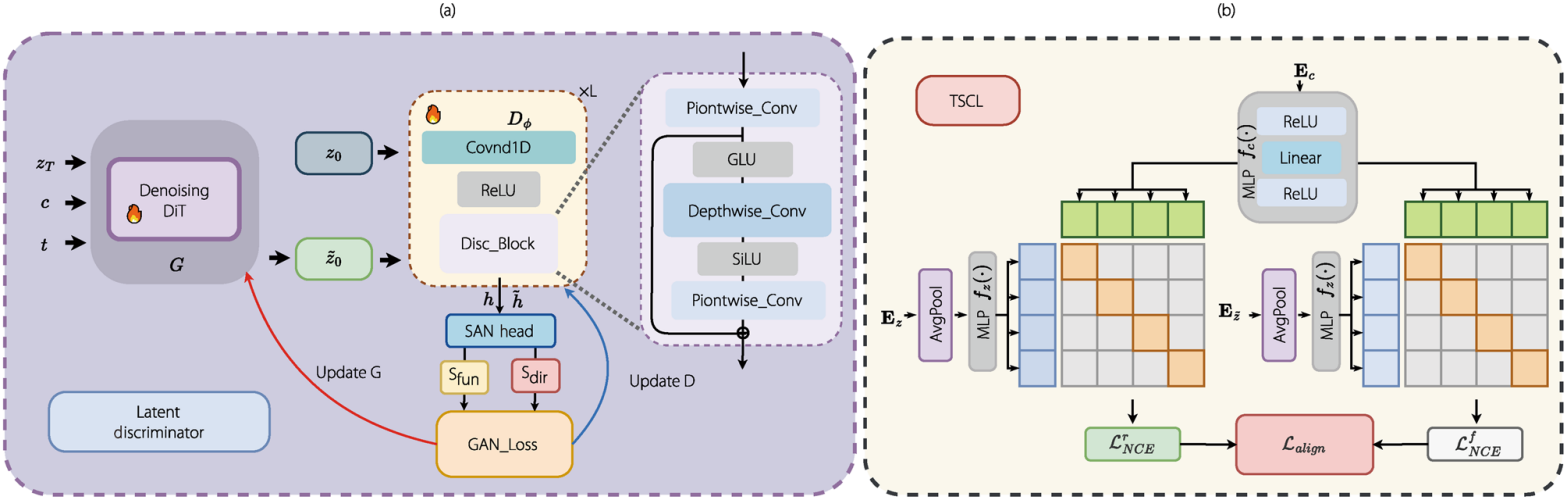
Adversarial motion latent discriminator and temporal semantic contrastive learning. **a** Structure of the latent discriminator; **b** Structure of the TSCL module. TSCL: Temporal semantic contrastive learning; DiT: Diffusion transformer

As illustrated in Fig. 2a, the proposed discriminator comprises a convolutional backbone and an SAN head [24]. The convolutional backbone is designed to extract temporal motion representations. Specifically, the real motion representations $\boldsymbol{z}_0$ and predicted representations $\tilde{\boldsymbol{z}}_0$ (generated by the denoising network conditioned on noise $\boldsymbol{z}_t$, text $c$, and timestep $t$) are considered as inputs. These representations are first projected via 1D convolution, followed by rectified linear unit activation for initial mapping. They are processed using a backbone composed of stacked disk blocks. Similar to depthwise separable convolutions, this architecture efficiently models both local motion details and long-range temporal dependencies. Finally, the representations for subsequent adversarial calculations are obtained: the real latent representations $h = D_\phi(\boldsymbol{z})$ and the generated latent representations $\tilde{h} = D_\phi(\tilde{\boldsymbol{z}}_0)$.

The latent motion representation inherently contains two orthogonal attributes, namely, dynamic intensity (e.g., velocity and magnitude), and motion direction (e.g., spatial pose and action category). Traditional convolutional layers tend to combine these two types of information. Consequently, in the early stages of training, the discriminator can easily be dominated by high-amplitude noise, leading to training instability.

To achieve fine-grained adversarial supervision, the authors introduce an SAN head at the end of the convolutional backbone. By performing an orthogonal decomposition on the kernel weights, the SAN head disentangles the feature flow into two distinct branches: a dynamic scale ($S_{fun}$) and a structural pose ($S_{dir}$). The dynamic-scale branch regulates the magnitude and velocity of the motion within the latent space. The authors employ hinge loss to stabilize the decision boundaries during training. The losses for the generator $G$ and discriminator $D_\phi$ are defined as follows:

3 $${\mathcal{L}_{fun}} = \left\{ {\begin{array}{*{20}{l}}{ - \mathbb{E}[{S_{fun}}(\tilde h)]}&{{\text{for }}G} \\ \begin{gathered}\mathbb{E}[\max (0,1 - {S_{fun}}(h))] \hfill \\+ \mathbb{E}[\max (0,1 + {S_{fun}}(\tilde h))] \hfill \\ \end{gathered} &{{\text{for }}{D_\phi }} \end{array}} \right.$$

The structural pose branch supervises the geometric structure, ensuring that the generated skeletal movements adhere to the authentic human kinematics. Linear loss is adopted to maximize the separability between the real and fake samples in the representation space:

4 $$\mathcal{L}_{dir} = \begin{cases} -\mathbb{E}[S_{dir}(\tilde{\boldsymbol{h}})] & \text{for } G \\ -\mathbb{E}[S_{dir}(\boldsymbol{h})] + \mathbb{E}[S_{dir}(\tilde{\boldsymbol{h}})] & \text{for } D_\phi \end{cases}$$

This design compels the generator to closely approximate the dynamic magnitude, while simultaneously recovering the motion structure within a disentangled feature space. The formulation of the training process is done as a min-max game between the generator $G$ (denoising network) and discriminator $D_\phi$. To ensure stability, the authors employ an alternating optimization strategy that updates the generator and discriminator sequentially at each iteration.

**Generator optimization.** The objective of generator $G$ is to synthesize motions capable of deceiving the discriminator. During this phase, the parameters of $D_\phi$ are frozen. The generator takes noise $\boldsymbol{z}_t$, text $c$, and timestep $t$ as inputs, and outputs the predicted sample $\tilde{\boldsymbol{z}}_0$. To ensure the generated motions approximate the real distribution in both dynamic scale and structural pose, the generator’s adversarial loss $\mathcal{L}_{adv}^{G}$ combines the two branch losses in a weighted manner:

5 $$\mathcal{L}_{adv}^{G} = \mathcal{L}_{fun}^{G} + \mathcal{L}_{dir}^{G}$$

By minimizing this loss, the generator is incentivized to produce samples that yield higher scores from the discriminator.

**Discriminator optimization.** The objective of the discriminator $D_\phi$ is to distinguish between real and generated motion sequences. During this phase, the parameters of generator $G$ are frozen. The discriminator receives both real latent representations $\boldsymbol{z}_0$ and generated latent representations $\tilde{\boldsymbol{z}}_0$ produced by the diffusion model. Specifically, by maximizing the scores for real samples and minimizing those for generated samples, a clear decision boundary is established between authentic and synthetic data. The final adversarial loss for the discriminator, $\mathcal{L}_{adv}^{D_\phi}$, is computed as the sum of losses for both real and generated samples:

6 $$\mathcal{L}_{adv}^{D_\phi} =\mathcal{L}_{fun}^{D_\phi} + \mathcal{L}_{dir}^{D_\phi}$$

By minimizing this loss, the discriminator enhances its precision when identifying physical attributes and structural representations. This process effectively mitigates the over-smoothing phenomenon common in diffusion generation, thereby improving physical realism and dynamic fidelity.

### TSCL

Although text embeddings $c$ are injected into the diffusion denoising backbone via attention mechanisms, this form of implicit semantic guidance is often insufficient for establishing precise text-motion mappings. In particular, when generating complex motions, the MSE loss focuses solely on the geometric reconstruction, neglecting the semantic consistency. Furthermore, although the adversarial discriminator effectively enhances dynamic realism, it primarily constrains the motion distribution, offering weak supervision over fine-grained semantics. To bridge this gap, inspired by multimodal contrastive learning (e.g., CLIP), the authors introduce an explicit TSCL (Fig. 2b). This strategy aims to construct a shared semantic manifold in which latent motion representations and their corresponding text embeddings are aligned as closely as possible within the vector space. Specifically, the module consists of three key steps: representation aggregation, joint space projection, and bidirectional contrastive optimization.

Because the latent motion representations $\boldsymbol{z}_0, \tilde{\boldsymbol{z}}_0 \in \mathbb{R}^{m \times d}$ are temporal sequences, and the text embedding $c$ is a global vector, first the temporal aggregation to compress the dynamic sequences into global representations is performed:

7 $$\boldsymbol{z} = \frac{1}{T} \sum_{t=1}^{T} \boldsymbol{z}_0^{(t)},\quad \tilde{\boldsymbol{z}} = \frac{1}{T} \sum_{t=1}^{T} \tilde{\boldsymbol{z}}_0^{(t)}$$

To eliminate the cross-modal heterogeneity, two lightweight projection networks, $f_c(\cdot)$ and $f_z(\cdot)$ (comprising fully connected layers and rectified linear unit activations) are designed. These map the aggregated real motion $\boldsymbol{z}$, generated motion $\tilde{\boldsymbol{z}}$, and text embeddings $\boldsymbol{c}$ onto a shared normalized contrastive space as follows:

8 $$\boldsymbol{e}_c = f_c(\boldsymbol{c}), \quad\boldsymbol{e}_{z} = f_z(\boldsymbol{z}),\quad\tilde{\boldsymbol{e}}_{z} = f_z(\tilde{\boldsymbol{z}})$$

Here, $\boldsymbol{e}_c, \boldsymbol{e}_z, \tilde{\boldsymbol{e}}_z \in \mathbb{R}^d$ represent the projected vectors for the text, real motion, and generated motion, respectively.

The authors employ InfoNCE to establish cross-modal semantic alignment. Given a batch of $N$ text-motion pairs, where $[{{\boldsymbol{E}}_{\boldsymbol{z}}} = \{ {\boldsymbol{e}}_{\boldsymbol{z}}^1, \ldots ,{\boldsymbol{e}}_{\boldsymbol{z}}^N\}$ and ${{\boldsymbol{\rm{E}}}_{\boldsymbol{\mathrm{c}}}} = \{ {\boldsymbol{\mathrm{e}}}_{\boldsymbol{\mathrm{c}}}^1, \ldots ,{\boldsymbol{\mathrm{e}}}_{\boldsymbol{\mathrm{c}}}^\mathrm{N}\}$ denote the feature sets for the two modalities, the loss for the $i$-th sample is defined as:

9 $$\mathcal{L}_{NCE}(\boldsymbol{e}_z^i, \boldsymbol{e}_c^i) = - \log \frac{\exp(\boldsymbol{e}_z^i \cdot \boldsymbol{e}_c^i / \tau)}{\sum_{j=1}^{N} \exp(\boldsymbol{e}_z^i \cdot \boldsymbol{e}_c^j / \tau)}$$

where $\tau$ is the temperature coefficient. The pair $(\boldsymbol{e}_z^i, \boldsymbol{e}_c^i)$ constitutes a positive sample, and the remaining $N-1$ combinations in the batch serve as negative samples. Furthermore, to prevent semantic drift during the denoising process, the authors utilize real samples as “semantic anchors” and ensure consistent alignment with the input text. The total alignment loss $\mathcal{L}_{align}$ is a weighted combination of two components as follows:

10 $$\mathcal{L}_{align} = \lambda_{r} \mathcal{L}_{NCE}^{r}(\boldsymbol{E}_{z}, \boldsymbol{E}_{c}) + \lambda_{f} \mathcal{L}_{NCE}^{f}(\boldsymbol{E}_{\tilde{z}}, \boldsymbol{E}_{c})$$

where $\boldsymbol{E}_c, \boldsymbol{E}_z, \boldsymbol{E}_{\tilde{z}}$ represent the projected feature sets for text, real motion, and generated motion within the current batch, respectively. $\lambda{r}$ and $\lambda_{f}$ denote the weights of the real and generated alignment terms, respectively. The real alignment term $\mathcal{L}_{NCE}^{r}$ acts as a regularizer, ensuring that projection network $f(\cdot)$ learns the correct real data distribution. Conversely, the generated alignment term $\mathcal{L}_{NCE}^{f}$ computes the contrastive loss between the predicted samples $\tilde{\boldsymbol{z}}_0$ and text. This dual contrastive strategy not only leverages negative supervision signals but also directly constrains the generator’s dynamic denoising process. This ensures an optimal balance between geometric realism (constrained by GAN) and semantic accuracy (constrained by contrastive loss).

### Training and inference

**Training objective.** The pre-trained VAE is treated as a frozen representation extractor. Real motion sequences are mapped into latent representations, $\boldsymbol{Z}_0 = \mathcal{E}(\boldsymbol{X})$ and use them to train the conditional diffusion model. During this phase, the diffusion denoising backbone, adversarial discriminator, and text-motion contrastive modules are jointly optimized. The total optimization objective for the generator, $\mathcal{L}_{total}$, is a weighted combination of the primary denoising, adversarial, and semantic alignment losses:

11 $$\mathcal{L}_{total} = \mathcal{L}_{LDM} + \lambda_{adv} \mathcal{L}_{adv}^{G} + \lambda_{align} \mathcal{L}_{align}$$

where $\lambda_{adv}$ and $\lambda_{align}$ denote the weights for the adversarial and contrastive terms, respectively. The discriminator is updated alternately by minimizing $\mathcal{L}_{adv}^{D_\phi}$. This multitask joint training mechanism ensures holistic improvements across geometric structures, dynamic details, and semantic consistency.

**Inference stage.** The authors sample the initial noise $\boldsymbol{z}_T$ from the standard Gaussian distribution $\mathcal{N}(\boldsymbol{0}, \boldsymbol{I})$. By utilizing the trained denoising network, the latent motion representations $\tilde{\boldsymbol{z}}_0$ are recovered through a $T$-step reverse denoising process. Finally, the target motion sequence is reconstructed using the decoder $\boldsymbol{X} = \mathcal{D}e(\tilde{\boldsymbol{z}}_0)$. Following the strategy established in MoLA [23], the authors employ classifier-free guidance to optimize the conditional generation process.

### Experiment datasets

The experimental assessment utilizes the two established datasets as described below. Following standard evaluation protocols, both sets are partitioned into training (80%), validation (15%), and testing (5%).

HumanML3D [1] contains 14,616 motion segments. Standard preprocessing for this dataset involves identifying clips of up to 10 s in duration and resampling them to 20 FPS. It contains 44,970 text annotations, 5371 words and an average sentence length of 12 words.

KIT-ML [25] is a relatively smaller dataset, consisting of 3911 motions, and downsampled to 12.5 FPS. This dataset includes 6278 textual annotations, averaging approximately 8 words in length, with a vocabulary size of 1,623.

### Evaluation metrics

The authors utilize the standard evaluation metrics proposed in T2M [1], including R-precision, FID, multimodal distance (MM-Dist), and diversity. FID measures the distributional distance between the generated and real motions, indicating geometric authenticity, whereas R-precision and MM-Dist assess how well the synthesized motions align semantically with the input text. Diversity measures the variance in the generated motions across the dataset. Unlike metrics for which higher or lower values are strictly better, a value closer to the diversity of the real dataset indicates better performance in terms of diversity. The ground-truth diversity values are 9.503 for HumanML3D and 11.08 for KIT-ML.

### Implementation details

The proposed motion encoder is built based on the architecture of MoLA [23]. This VAE compresses raw motion sequences into continuous latent representations, with dimensions of $d=16$. The denoising backbone used adopts a diffusion transformer-based decoder architecture for the diffusion process within the latent space. It is configured with nine layers, four attention heads, and 512 hidden dimensions. The authors utilize a pre-trained CLIP ViT-L/14 as the text encoder, which remains frozen throughout the training phase. The projection heads for both discriminator and contrastive alignments are set to dimensions of 1024. In the contrastive learning module, the weights for the real and generated alignment terms are set to $\lambda_{r}=1$ and $\lambda_{f}=0.1$, respectively. The authors employ the AdamW optimizer for end-to-end training with a batch size of 64. For the HumanML3D dataset, the learning rate is set to $10^{-4}$ for 100,000 iterations. The adversarial loss weight is set to $\lambda_{adv}=1 \times 10^{-4}$, while the contrastive alignment weight is set to $\lambda_{align}=0.03$. To prevent overfitting for the smaller KIT-ML dataset, the learning rate is reduced to $5 \times 10^{-5}$ and training duration to 40,000 iterations. Accordingly, the adversarial weight is adjusted to $\lambda_{adv}=1 \times 10^{-3}$ and the contrastive weight to $\lambda_{align}=0.01$. All the experiments are conducted on a single NVIDIA V100 GPU.

## Results and Discussion

### Quantitative comparison

Tables 1 and 2 present the quantitative results on the two datasets, respectively. In general, for the primary HumanML3D benchmark, the proposed method outperforms existing diffusion-based methods in terms of motion quality and semantic consistency. Notably, it surpasses VQ-based methods on key metrics, demonstrating the effectiveness of the proposed enhancements in latent-space diffusion modeling.

**Table 1:** Performance comparison on the HumanML3D dataset

Method	Framework	**R-precision**$\uparrow$			**FID**$\downarrow$	**MM-Dist**$\downarrow$	**Diversity**$\rightarrow$
		Top-1	Top-2	Top-3			
Real motion		$0.511{\:\pm\:0.003}$	$0.703{\:\pm\: 0.003}$	$0.797{\:\pm\:0.002}$	$0.002{\:\pm\:0.000}$	$2.974{\:\pm\:0.008}$	$9.503{\:\pm\:0.065}$
MMM [26]	Vector quantization	$0.504{\:\pm\:0.003}$	$0.696{\:\pm\:0.003}$	$0.794{\:\pm\:0.002}$	$0.080{\:\pm\:0.003}$	$2.998{\:\pm\:0.007}$	$9.411{\:\pm\:0.058}$
MoMask [27]		$0.521{\:\pm\:0.002}$	$0.713{\:\pm\:0.002}$	$0.807{\:\pm\:0.002}$	$\boldsymbol{0.045}{\:\pm\:0.002}$	$2.958{\:\pm\:0.008}$	–
MDM [4]	Diffusion	$0.320{\:\pm\:0.005}$	$0.498{\:\pm\:0.004}$	$0.611{\:\pm\:0.007}$	$0.544{\:\pm\:0.044}$	$5.566{\:\pm\:0.027}$	$9.559{\:\pm\:0.086}$
MLD [7]		$0.481{\:\pm\:0.003}$	$0.673{\:\pm\:0.003}$	$0.772{\:\pm\:0.002}$	$0.473{\:\pm\:0.013}$	$3.196{\:\pm\:0.010}$	$9.724{\:\pm\:0.082}$
MARDM [9]		$0.500{\:\pm\:0.004}$	$0.695{\:\pm\:0.003}$	$0.795{\:\pm\:0.003}$	$0.114{\:\pm\:0.007}$	$3.270{\:\pm\:0.009}$	–
Fg-T2M++ [28]		$0.513{\:\pm\:0.002}$	$0.702{\:\pm\:0.002}$	$0.801{\:\pm\:0.003}$	$0.089{\:\pm\:0.004}$	$\boldsymbol{2.925{\:\pm\:0.007}}$	$9.223{\:\pm\:0.114}$
MoLA [23]		$0.516{\:\pm\:0.006}$	$0.712{\:\pm\:0.005}$	$0.805{\:\pm\:0.004}$	$0.115{\:\pm\:0.004}$	$3.008{\:\pm\:0.016}$	$9.885{\:\pm\:0.152}$
**Ours**	**Diffusion**	$\boldsymbol{0.523}{\:\pm\:0.002}$	$\boldsymbol{0.718}{\:\pm\:0.002}$	$\boldsymbol{0.810}{\:\pm\:0.002}$	$\underline{0.067}{\:\pm\:0.003}$	$\underline{2.943{\:\pm\:0.006}}$	$\boldsymbol{9.519}{\:\pm\:0.082}$

Bold highlights the best results, and underline values mark the second-best. – indicates that the corresponding results were not reported in the original literature. The symbols $\downarrow$, $\uparrow$, and $\rightarrow$ correspond to metrics where lower, higher, or values closer to real data indicate better performance. For each metric, we repeat the evaluation 20 times and report the average with 95%CI. FID: Fréchet inception distance; MM-Dist: Multimodal distance

**Table 2:** Quantitative evaluation on the KIT-ML test set

Method	Framework	**R-precision**$\uparrow$			**FID**$\downarrow$	**MM-Dist**$\downarrow$	**Diversity**$\rightarrow$
		Top-1	Top-2	Top-3			
Real motion		$0.424{\:\pm\:0.005}$	$0.649{\:\pm\:0.006}$	$0.779{\:\pm\:0.006}$	$0.031{\:\pm\:0.004}$	$2.788{\:\pm\:0.012}$	$11.08{\:\pm\:0.097}$
MMM [26]	Vector quantization	$0.404{\:\pm\:0.005}$	$0.621{\:\pm\:0.005}$	$0.744{\:\pm\:0.004}$	$0.316{\:\pm\:0.028}$	$2.977{\:\pm\:0.019}$	$10.91{\:\pm\:0.101}$
MoMask [27]		$0.433{\:\pm\:0.007}$	$0.656{\:\pm\:0.005}$	$0.781{\:\pm\:0.005}$	$0.204{\:\pm\:0.011}$	$2.779{\:\pm\:0.022}$	–
MDM [4]	Diffusion	$0.164{\:\pm\:0.004}$	$0.291{\:\pm\:0.004}$	$0.396{\:\pm\:0.004}$	$0.497{\:\pm\:0.021}$	$9.191{\:\pm\:0.022}$	$10.847{\:\pm\:0.109}$
MLD [7]		$0.390{\:\pm\:0.008}$	$0.609{\:\pm\:0.008}$	$0.734{\:\pm\:0.007}$	$0.404{\:\pm\:0.027}$	$3.204{\:\pm\:0.027}$	$10.80{\:\pm\:0.117}$
MARDM [9]		$0.387{\:\pm\:0.006}$	$0.610{\:\pm\:0.006}$	$0.749{\:\pm\:0.006}$	$0.242{\:\pm\:0.014}$	$3.374{\:\pm\:0.019}$	–
Fg-T2M++ [28]		$\boldsymbol{0.442}{\:\pm\:0.006}$	$0.657{\:\pm\:0.005}$	$0.781{\:\pm\:0.004}$	$\boldsymbol{0.135}{\:\pm\:0.004}$	$\boldsymbol{2.696}{\:\pm\:0.011}$	$\boldsymbol{10.99}{\:\pm\:0.105}$
MoLA [23]		$0.432{\:\pm\:0.008}$	$0.655{\:\pm\:0.008}$	$0.770{\:\pm\:0.004}$	$0.529{\:\pm\:0.056}$	$2.942{\:\pm\:0.053}$	$11.129{\:\pm\:0.158}$
**Ours**	**Diffusion**	$0.439{\:\pm\:0.007}$	$\boldsymbol{0.675}{\:\pm\:0.006}$	$\boldsymbol{0.798}{\:\pm\:0.004}$	$0.334{\:\pm\:0.022}$	$2.793{\:\pm\:0.018}$	$10.940{\:\pm\:0.082}$

Bold highlights the best results, and underline values mark the second-best. – indicates that the corresponding results were not reported in the original literature. The symbols $\downarrow$, $\uparrow$, and $\rightarrow$ correspond to metrics where lower, higher, or values closer to real data indicate better performance. For each metric, we repeat the evaluation 20 times and report the average with 95%CI. FID: Fréchet inception distance; MM-Dist: Multimodal distance

**Motion quality.** The proposed method significantly outperforms others in the FID metric, achieving a score of 0.067 on HumanML3D. Compared to standard diffusion baselines, such as MDM [4] and MLD [7], the proposed approach yields a substantially lower FID score. This improvement is primarily attributed to the local motion modeling module and the adversarial discriminator. The former alleviates the ‘over-smoothing’ issue common in diffusion models, while the latter constrains the generated motions to lie on the authentic motion manifold, thereby eliminating unnatural jitter. Furthermore, unlike VQ-based methods plagued by quantization errors, the continuous latent-space modeling preserves fine-grained motion textures.

**Semantic alignment.** The proposed model performs strongly on R-precision and MM-Dist metrics that assess text-motion consistency. The use of internal contrastive alignment mechanism achieves superior semantic matching. This validates the effectiveness of explicitly optimizing the mutual information within a latent space to capture complex textual instructions.

### Qualitative comparison

**Fig. 3:**
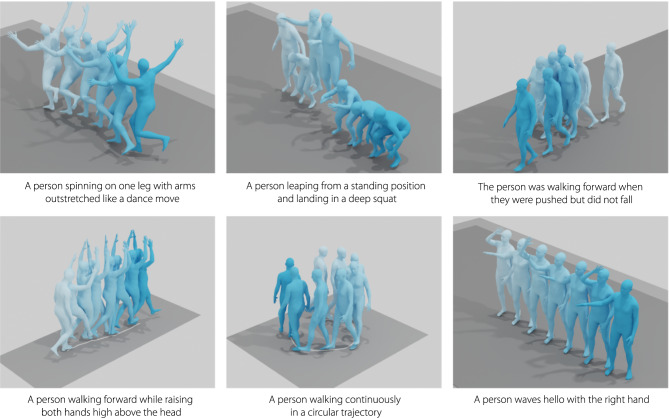
Qualitative comparison of generated motions given diverse text descriptions,including simple, compositional, and disturbance scenarios

To further evaluate the effectiveness of the proposed method, the authors first present qualitative results in Fig. 3, covering a diverse set of motion descriptions, including simple actions (e.g., “waving hello with the right hand”), compositional motions (e.g., “walking forward while raising both hands”), and more challenging scenarios involving disturbances (e.g., “walking forward when they were pushed but did not fall”). From these examples, it can be observed that the proposed model can generate motions that closely align with the input text semantics. In particular, it demonstrates a strong capability to capture fine-grained details and handle compositional descriptions, producing temporally coherent and physically plausible motion sequences.

**Fig. 4:**
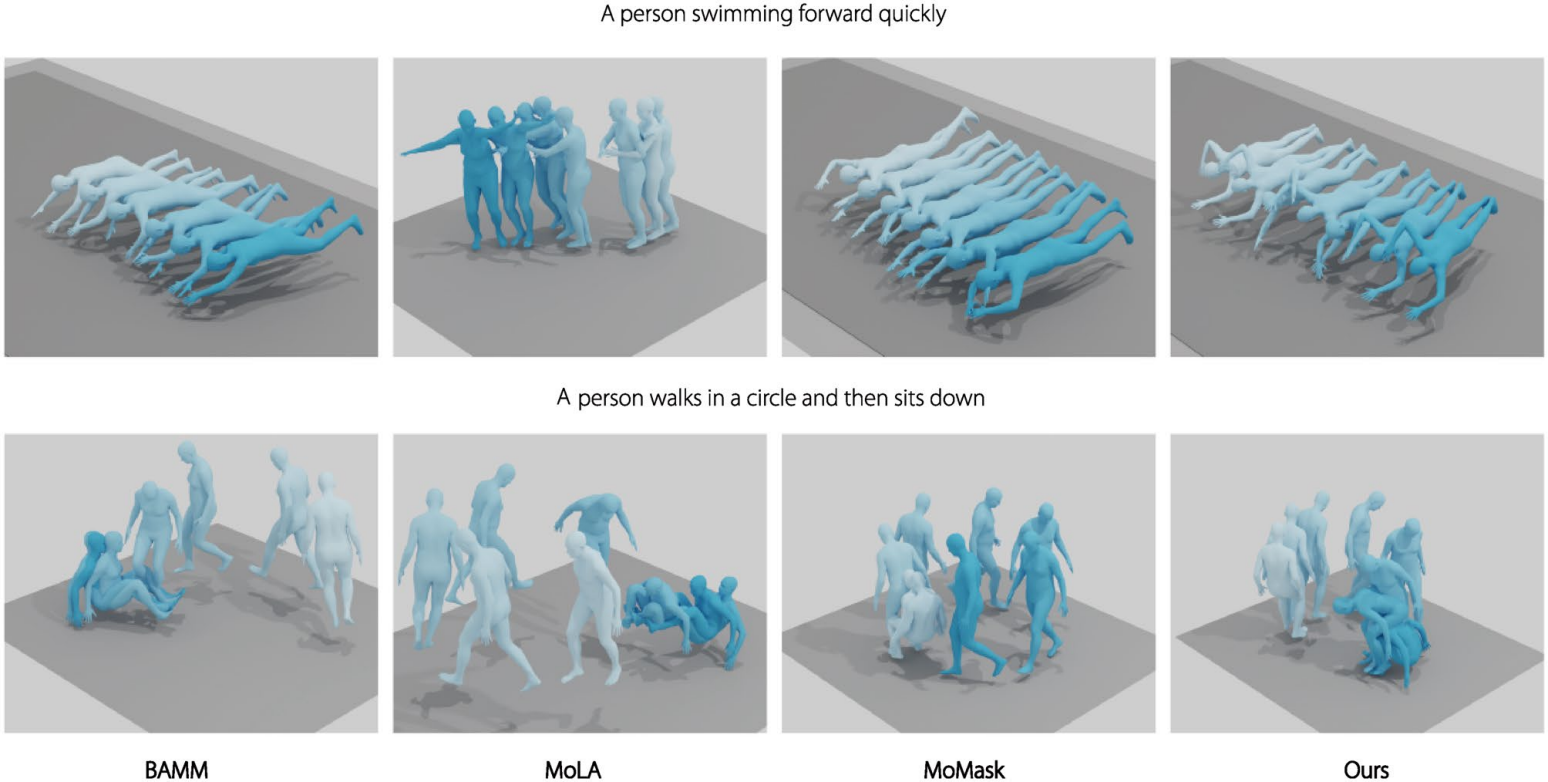
Visual comparison of text-driven motion generation. The proposed method is compared against BAMM, MoLA, and MoMask. The results demonstrate that the proposed method generates motion sequences with higher semantic accuracy and more natural trajectories

Complementing these observations, the authors select representative SOTA methods from both diffusion-based and VQ-based paradigms for comprehensive comparison. Visual comparisons with BAMM [29], MoLA [23], and MoMask [27] methods are provided. Figure 4 shows a qualitative visualization of the generated motions. Visually, baseline methods often encounter semantic misalignment or motion blurring when processing complex or lengthy textual descriptions, whereas the proposed model synthesizes sequences that exhibit superior temporal coherence and richer details. For instance, in sequences involving intricate hand movements or rapid transitions, the proposed method demonstrates superior physical realism, effectively mitigating common artifacts such as foot sliding and unnatural joint distortions. This improvement in the visual quality corroborates the exceptionally low FID scores achieved.

### User study

**Fig. 5:**
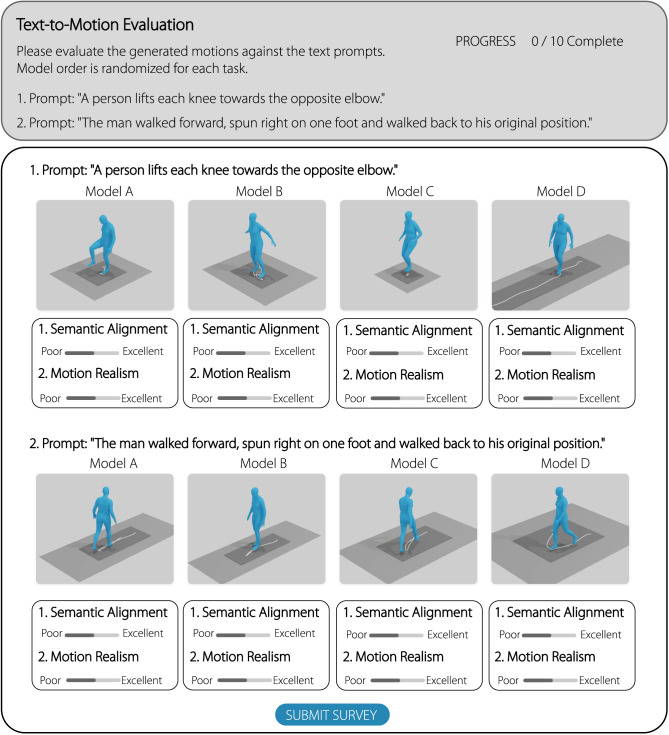
Interface of the user study for text-to-motion evaluation. Participants rategenerated motions from different models based on semantic alignment and motionrealism under the same text prompts

A user study is conducted to evaluate the generated motions in terms of human perception as shown in Fig. 5. The participants are asked to rate the results of different methods (BAMM [29], MoLA [23], MoMask [27], and the proposed method) using the same text prompts, focusing on semantic alignment and motion realism. The order of the models was randomized to avoid bias.

**Fig. 6:**
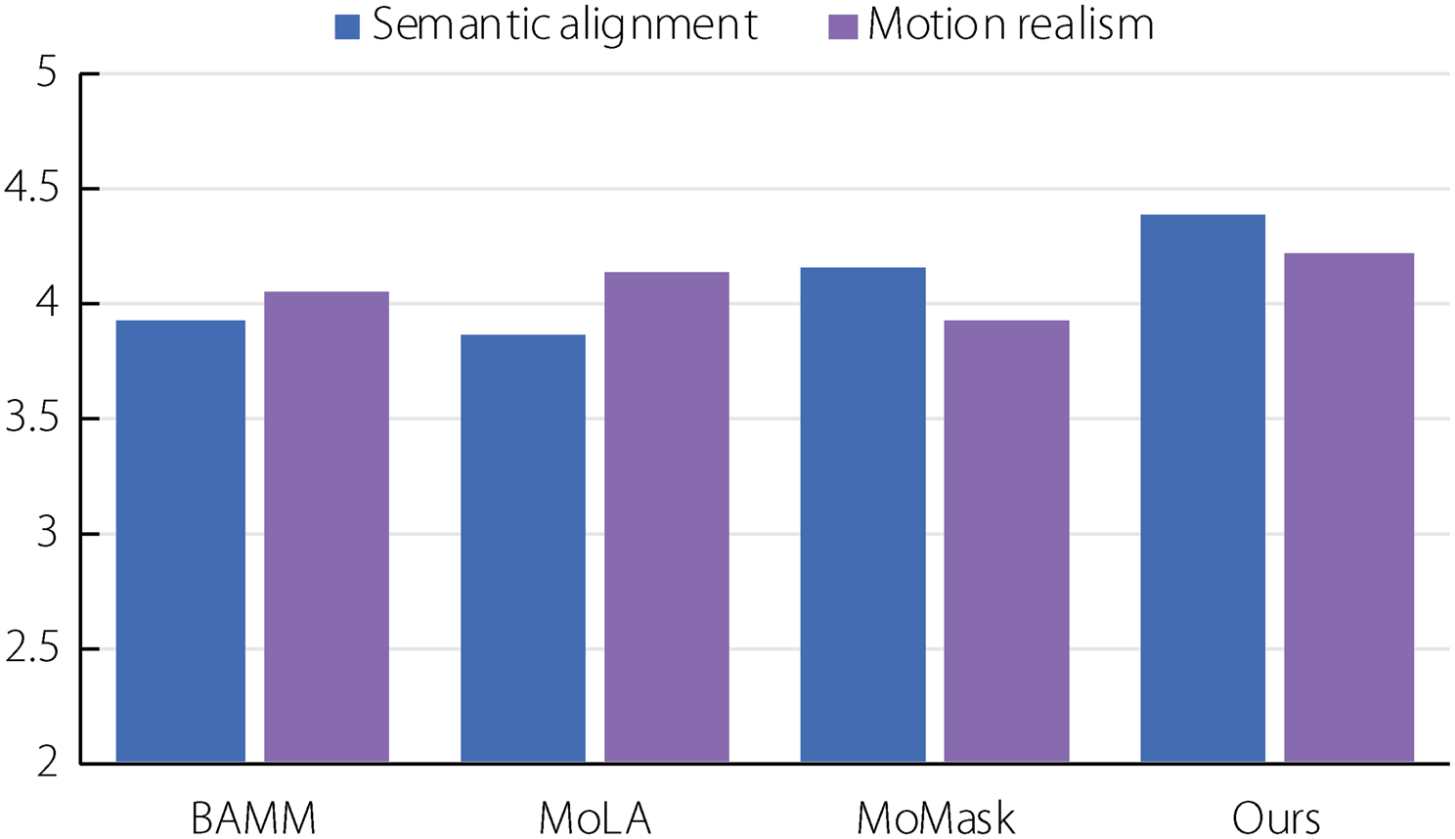
Results of the user study. The proposed method achieves higher scores in bothsemantic alignment and motion realism compared to baseline methods

As shown in Fig. 6, the proposed method achieves the highest scores on both abovementioned criteria, indicating better consistency with the input text and more natural motion quality. This observation is consistent with the quantitative and qualitative results.

### Ablation experiment

Ablation studies were performed on HumanML3D to evaluate the role of each module and the effects of the major hyperparameters.

**Impact of individual components.** To isolate the specific contribution of each component to the overall performance, a comprehensive ablation study is conducted on the HumanML3D dataset, with quantitative results summarized in Table 3. Each proposed module enhances the final model’s performance across distinct dimensions.

**Table 3:** Quantitative ablation results on HumanML3D

Method	**FID**$\downarrow$	**R-precision**$\uparrow$			**MM-Dist**$\downarrow$	**Diversity**$\rightarrow$
		Top-1	Top-2	Top-3		
Baseline (MoLA)	0.115	0.516	0.712	0.805	3.008	9.885
w/Disc	0.081	0.513	0.712	0.805	2.999	9.593
w/Contrastive	0.094	0.523	0.715	0.808	2.952	9.598
Full model	**0.067**	**0.523**	**0.718**	**0.810**	**2.943**	**9.519**

The best values are highlighted in bold. The symbols ↓, ↑, and → correspond to metrics where lower, higher, or values closer to real data indicate better performance. FID: Fréchet inception distance; MM-Dist: Multimodal distance

**Effect of the adversarial discriminator.** Upon removing the adversarial discriminator, the FID score significantly deteriorates (rising from 0.067 to 0.081), suggesting that the absence of a distribution-level constraint compromises the naturalness of the generated motions. Although the R-precision remains relatively stable, a slight increase in diversity (from 9.519 to 9.593) implies that the generated motions become more unconstrained and deviate from the authentic distribution. This underscores the critical role of discriminators in ensuring the realism of the global motion.

**Effect of the contrastive alignment.** Excluding the cross-modal contrastive learning leads to a comprehensive decline in R-precision (Top-3 drops from 0.810 to 0.808), indicating compromised semantic consistency. Simultaneously, the FID increases from 0.067 to 0.094, revealing that insufficient semantic alignment indirectly impairs generation quality. This observation aligns with the authors’ hypothesis that when textual representations fail to constrain the latent sampling process effectively, the diffusion chain becomes prone to semantic drift, causing the generated results to diverge from the textual description.

The full model achieves optimal performance across all metrics, with FID improving to 0.067 and R-precision peaking at 0.523/0.718/0.810. It significantly outperforms the other configurations in terms of motion naturalness and semantic alignment. This demonstrates the complementarity of the proposed components. The discriminator ensures global dynamic realism, whereas contrastive alignment reinforces semantic consistency, collectively enhancing the generative capabilities of the diffusion framework.

**Fig. 7:**
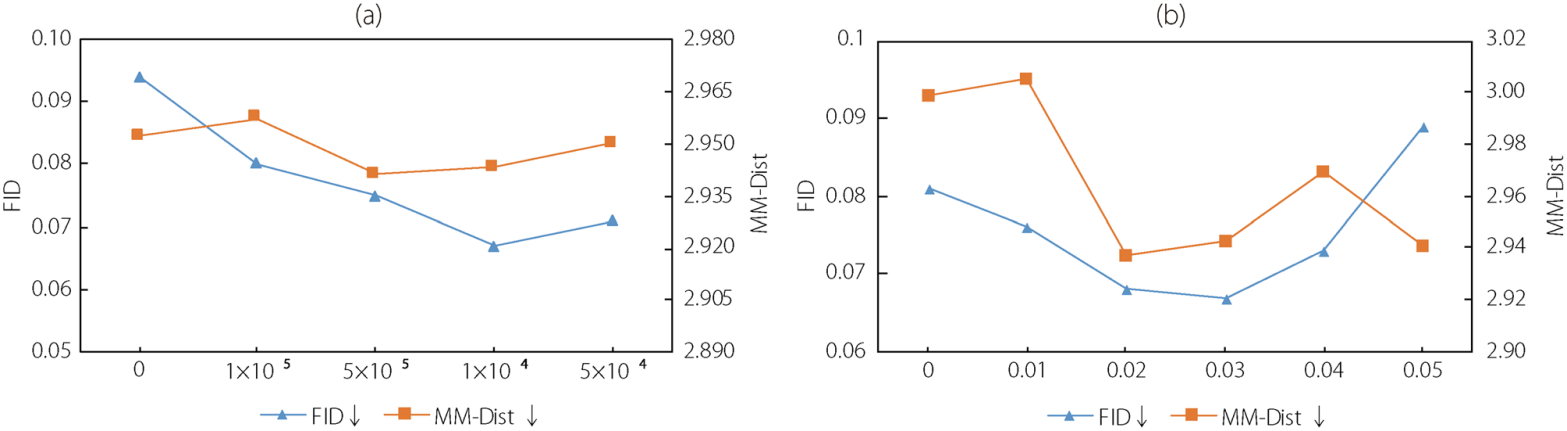
Analysis of adversarial and contrastive loss weights. **a** Effect of the adversarial weight $\lambda_{\mathrm{adv}}$ on FID and MM-Dist. The horizontal axis uses manually selected discrete hyperparameter values (0, 1e-5, 5e-5, 1e-4, 5e-4) with non-uniform spacing for comparative analysis; **b** Effect of the contrastive loss weight $\lambda_{\mathrm{align}}$ on FID and MM-Dist. FID: Fréchet inception distance; MM-Dist: Multimodal distance

**Sensitivity to adversarial weight **$\lambda_{adv}$. The impact of the adversarial loss weight $\lambda_{\mathrm{adv}}$ on generation performance is further investigated. Because the discriminator operates in latent motion space, only a weak adversarial signal is required to regularize the diffusion process. As shown in Fig. 7a, the results demonstrate that increasing $\lambda_{adv}$ initially leads to a consistent reduction in FID, indicating that moderate adversarial supervision effectively alleviates the over-smoothing effect induced by the MSE-based diffusion objective and promotes more realistic motion generation. However, when $\lambda_{adv}$ exceeds $1\times10^{-4}$, both FID and MM-Dist begin to deteriorate. This suggests that excessively strong adversarial constraints may interfere with diffusion training dynamics, resulting in degraded distribution quality and weakened text-motion alignment. Based on this trade-off, $\lambda_{adv} = 1\times10^{-4}$ is adopted in all experiments, which achieves a favorable balance between physical realism and semantic consistency.

**Sensitivity analysis of contrastive loss weight **$\lambda_{align}$. The effect of contrastive alignment weight $\lambda_{align}$ on motion quality and text-motion consistency is studied. As shown in Fig. 7b, increasing $\lambda_{align}$ from 0 to 0.03 consistently improves the generation quality, as reflected by a lower FID score, indicating that moderate contrastive supervision effectively enhances semantic grounding during diffusion. When $\lambda_{align}$ exceeds 0.03, the FID score begins to increase, and MM-Dist shows noticeable fluctuations, suggesting that overly strong alignment constraints may limit the model’s flexibility in capturing fine-grained motion dynamics. Therefore, $\lambda_{align} = 0.03$ is adopted as the default setting, which provides a favorable balance between motion realism and text-motion consistency.

**Fig. 8:**
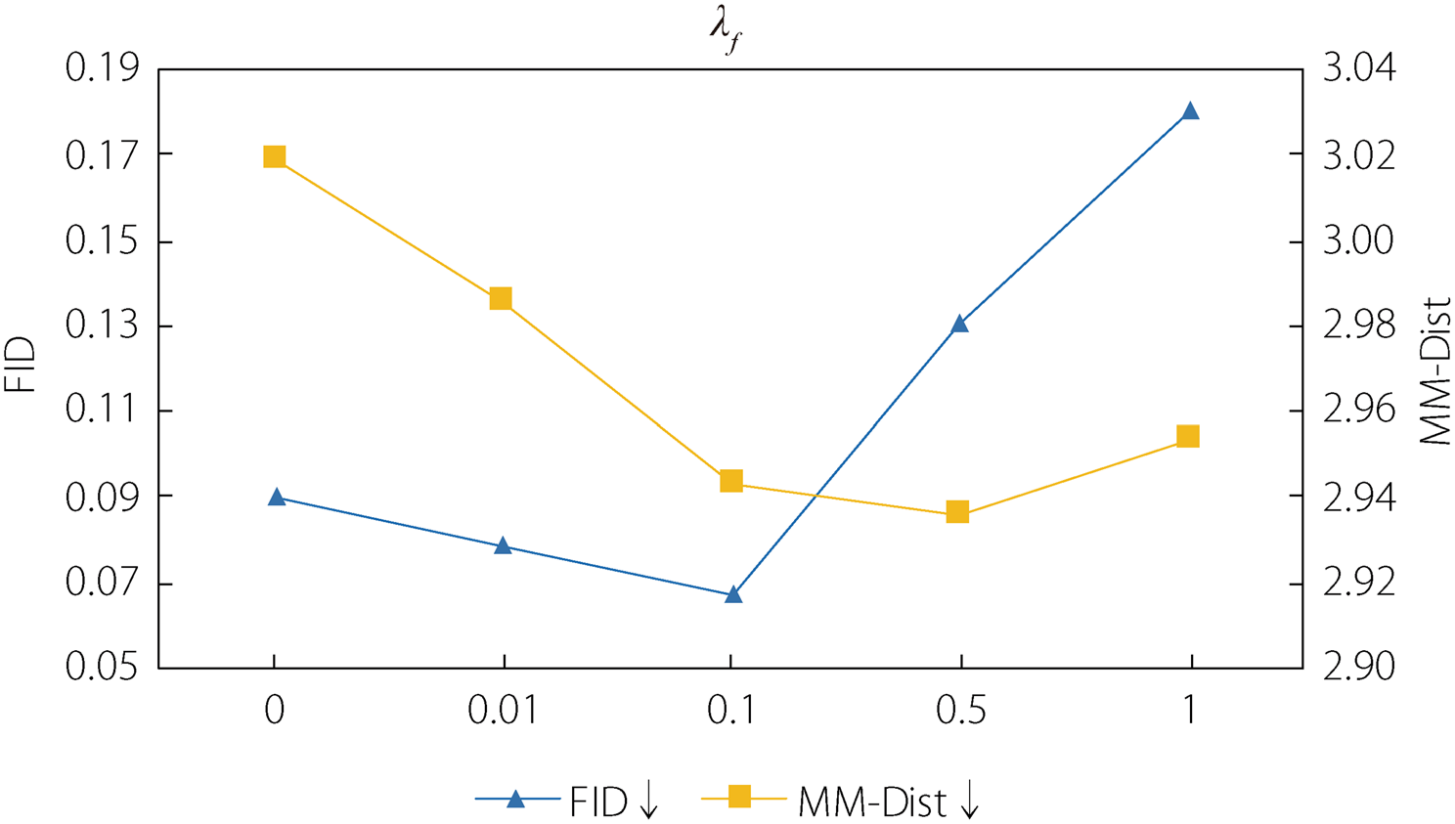
Analysis of alignment weights. $\lambda_{\mathrm{f}}$ on FID and MM-dist. The horizontal axis uses manually selected discrete hyperparameter values (0, 0.01, 0.1, 0.5, 1) with non-uniform spacing for comparative analysis. FID: Fréchet inception distance; MM-Dist: Multimodal distance

**Sensitivity to alignment weights.** Within the contrastive alignment module, the real sample alignment term $\lambda_{r}$ is used as a semantic anchor, and the generated sample alignment term $\lambda_{f}$ as an optimization guide. To investigate the optimal balance between these components, $\lambda_{r}=1.0$ is fixed and the impact of varying the intensity of $\lambda_{f}$ on the model’s performance is evaluated, as illustrated in Fig. 8. The results indicate that completely removing the generated alignment $\lambda_{f}=0$ results in a significant deterioration in MM-Dist, thereby validating the necessity of explicit semantic guidance. However, an excessive weight (e.g., $\lambda_{f}=1.0$) while maintaining high semantic matching results in the degradation of the FID score. This suggests that overly rigid semantic constraints may compromise the naturalness of the motion distribution. Ultimately, $\lambda_{f}=0.1$ is adopted as a setting that achieves optimal semantic consistency while preserving high-fidelity motion generation.

## Conclusions

This study addresses critical challenges in text-driven human motion generation, specifically the issues of insufficient semantic consistency and distribution shifts inherent in diffusion models. The study proposes a systematic framework for enhancements tailored to latent-space diffusion. Leveraging the expressive power of continuous latent spaces, the authors strategically integrate two core components, namely, an adversarial motion discriminator and a TSCL mechanism. In particular, the adversarial motion discriminator enhances the realism and dynamic naturalness of the motion distribution within the latent space. The TSCL explicitly enforces semantic consistency by injecting cross-modal alignment signals throughout diffusion. Extensive evaluations demonstrate that the proposed framework achieves significant improvements in the reported metrics. Furthermore, the enhanced diffusion model delivers a performance comparable to that of SOTA VQ-based approaches, underscoring the substantial potential of continuous diffusion frameworks for future motion generation tasks.

Despite these advances, the proposed approach has several limitations. The iterative sampling nature of diffusion models still incurs computational overhead during inference. In addition, the introduction of adversarial and contrastive objectives adds complexity to the optimization process during training.

Future research avenues include exploring mechanisms that explicitly model physical feasibility and environmental interactions, ensuring that the generated motions remain physically plausible across varying terrains, force conditions, and scene constraints.

## Data Availability

The datasets used in this study, HumanML3D and KIT-ML, are publicly availabledatasets.

## Abbreviation

VQ: Vector quantization
SOTA: State-of-the-art
MSE: Mean squared error
GAN: Generative adversarial network
CLIP: Contrastive language-image pre-training
VAE: Variational autoencoder
TSCL: Temporal semantic contrastive learning
infoNCE: Information noise-contrastive estimation
LDM: Latent diffusion model
DiT: Diffusion transformer
KL: Kullback-Leibler divergence
FID: Fréchet inception distance
MM-Dist: Multimodal distance

## Code availability

Available on https://excelsior-w.github.io/ARDT/.
